# INTEROBSERVER AGREEMENT OF INTRAPAPILLARY CAPILLARY LOOPS CLASSIFICATION FOR SUPERFICIAL ESOPHAGEAL SQUAMOUS CELL CARCINOMA IN A WESTERN CENTER

**DOI:** 10.1590/S0004-2803.24612024-120

**Published:** 2025-09-05

**Authors:** Fauze MALUF-FILHO, Ossamu OKAZAKI, Beanie Conceição Medeiros NUNES, Adriana Vaz SAFATLE-RIBEIRO, Luciano LENZ, Bruno Costa MARTINS

**Affiliations:** ¹ Faculdade de Medicina da Universidade de São Paulo, Departamento de Gastroenterologia, São Paulo, SP, Brasil.; 2 Instituto do Câncer, ICESP-HCFMUSP, São Paulo, SP, Brasil.

**Keywords:** Intrapapillary capillary loop, early esophageal cancer, interobserver agreement, intraobserver agreement, magnifying endoscopy with narrow-band imaging, superficial esophageal neoplasm, Capilares intrapapilares, câncer precoce de esôfago, concordância interobservador, concordância intraobservador, endoscopia com magnificação e narrow-band imaging, neoplasia superficial de esôfago

## Abstract

**Background:**

Accurate evaluation of the invasion depth of superficial esophageal squamous cell carcinoma (SESCC) is crucial for optimal treatment. While magnifying endoscopy (ME) using the Japanese Esophageal Society (JES) classification is reported as the most accurate method to predict invasion depth, its efficacy has not been tested in the Western world. This study aims to evaluate the interobserver agreement of the JES classification for SESCC and its accuracy in estimating invasion depth in a Brazilian tertiary hospital.

**Methods:**

We retrospectively selected ME with Narrow Band Imaging (ME-NBI) images of 30 suspected SESCC cases. The best images of each case were included in online forms, which were evaluated by ten endoscopists (five experts and five novices). The evaluators classified the lesions according to the JES-IPCL classification and estimated the depth of invasion. Interobserver agreement was assessed using kappa values. Histological comparison was possible for 17 lesions.

**Results:**

The overall interobserver agreement for the JES-IPCL classification was moderate (K=0.455, *P*<0.001). Agreement among experts (K=0.437) and novices (K=0.483) was also moderate. Sensitivity, specificity, and accuracy for IPCL types were: B1 (41.3%, 78.9%, 59.9%), B2 (75%, 66.7%, 68.7%), and B3 (46%, 91.7%, 78.6%). Overall accuracy of the JES classification for estimating depth of invasion was 47.5%.

**Conclusion:**

The moderate interobserver agreement suggests the JES-IPCL classification may be useful in the Western world, but extensive training is needed. The findings indicate a longer learning curve for accurate ME-NBI image evaluation using the JES classification.

## HIGHLIGHTS

• The study evaluates the JES classification for SESCC in a Brazilian hospital.

• Interobserver agreement was moderate (k=0.455), similar for experts and novices.

• Type B2 had the highest sensitivity (75%), but overall accuracy was 47.5%.

• Extensive training is needed for accurate ME-NBI image evaluation.

## RESUMO

**Contexto:**

A avaliação precisa da profundidade de invasão do carcinoma espinocelular superficial de esôfago (SESCC) é crucial para o tratamento ideal. Embora a endoscopia com magnificação (ME) utilizando a classificação da Sociedade Japonesa de Esôfago (JES) seja considerada o método mais preciso para prever a profundidade de invasão, sua eficácia não foi testada no mundo ocidental. Este estudo tem como objetivo avaliar a concordância interobservador da classificação JES para SESCC e sua precisão em estimar a profundidade de invasão em um hospital terciário brasileiro.

**Métodos:**

Selecionamos retrospectivamente imagens de ME com Narrow Band Imaging (ME-NBI) de 30 casos suspeitos de SESCC. As melhores imagens de cada caso foram incluídas em formulários online, que foram avaliados por dez endoscopistas (cinco especialistas e cinco iniciantes). Os avaliadores classificaram as lesões de acordo com a classificação JES-IPCL e estimaram a profundidade de invasão. A concordância interobservador foi avaliada utilizando os valores de kappa. A comparação histológica foi possível em 17 lesões.

**Resultados:**

A concordância interobservador geral para a classificação JES-IPCL foi moderada (K=0,455, *P*<0,001). A concordância entre especialistas (K=0,437) e iniciantes (K=0,483) também foi moderada. Sensibilidade, especificidade e precisão para os tipos IPCL foram: B1 (41,3%, 78,9%, 59,9%), B2 (75%, 66,7%, 68,7%) e B3 (46%, 91,7%, 78,6%). A precisão geral da classificação JES para estimar a profundidade de invasão foi de 47,5%.

**Conclusão:**

A concordância interobservador moderada sugere que a classificação JES-IPCL pode ser útil no mundo ocidental, mas é necessário treinamento extenso. Os achados indicam uma curva de aprendizado mais longa para a avaliação precisa de imagens de ME-NBI utilizando a classificação JES.

## INTRODUCTION

Esophageal squamous cancer (ESCC) is a malignant tumor that arises from the squamous epithelium lining the esophagus. ESCC is the seventh most common cancer and the sixth leading cause of cancer-related deaths worldwide^1^, particularly prevalent in areas with a high prevalence of tobacco and alcohol consumption. Early detection and accurate staging of ESCC are crucial for optimal treatment outcomes.

Endoscopy with magnification and evaluation of intrapapillary capillary loops (IPCL) has emerged as a valuable technique in the diagnosis and management of esophageal squamous cell carcinoma (ESCC)^2^. IPCL evaluation refers to the assessment of the tiny blood vessels within the surface layer of the esophageal mucosa, which undergo characteristic changes in ESCC. IPCL evaluation is performed using magnifying endoscopy, which involves the use of specialized endoscopes capable of providing high-resolution images of the esophageal mucosa. Endoscopy procedure incorporate optical zoom capabilities, allowing for detailed visualization of the tissue surface. By magnifying the image, endoscopists can identify subtle morphological changes in the IPCLs that can be indicative of ESCC. In normal esophageal mucosa, IPCLs appear as thin, straight vessels arranged in a regular network pattern. However, in ESCC, these vessels undergo characteristic changes. 

The IPCL patterns observed in ESCC include dilation, tortuosity, irregular branching, and microvascular density. These changes are thought to be a result of neo angiogenesis, which is the formation of new blood vessels to support the growing tumor. The identification and characterization of IPCL patterns in ESCC can provide important diagnostic and prognostic information^3^ ^-^ ^5^. Studies have shown that certain IPCL patterns are highly specific for ESCC and can differentiate it from benign lesions or other types of esophageal cancer.

Additionally, the severity and extent of IPCL changes have been correlated with tumor stage and prognosis, with more advanced stages exhibiting more pronounced alterations. However, the interpretation of these patterns can vary among observers due to different levels of experience and training. Several studies have shown that the agreement among observers in the classification of IPCL can range from moderate to substantial, depending on the expertise and familiarity of the observers with specific vascular patterns associated with different stages of ESCC^6^. Additionally, proper training and experience in interpreting IPCL patterns also play an important role in reducing variability among observers. Practice and continuous study of IPCL vascular patterns can help observers develop stronger skills and knowledge in the classification and interpretation of these patterns. Therefore, while there is some variation among observers in the classification of IPCL, the use of standardized systems and the enhancement of experience and training can help reduce this variability and improve consistency in the interpretation of IPCL vascular patterns^7^.

We aimed to evaluate the interobserver agreement of the JES classification of esophageal IPCL for superficial ESSC and its accuracy in estimate the depth of invasion, in a western cancer center.

## METHODS

### Study design

The study protocol was approved by the Institutional Review Board (IRB) of our institution (registration number: NP038/14). Compliance with ethical regulations and research standards was ensured, including obtaining informed consent from patients for the use of their images.

### Study population

The study included images from 30 lesions suspected of being esophageal squamous cell carcinoma (ESCC), examined with magnifying endoscopy and narrow band imaging (ME-NBI) between January 2011 and July 2018. The objective was to evaluate the interobserver agreement of the Japan Esophageal Society (JES) classification system. The still images were selected by two senior endoscopists who did not participate in the final classification process.

### Endoscopic procedure

Endoscopic examinations were performed using a magnifying endoscope (GIF-Q160Z, Olympus Medical System Co., Tokyo, Japan) with a dedicated hood attached to the tip. Patients were sedated with midazolam, fentanyl, and propofol. After thorough cleaning with a defoamer solution (simethicone), ME-NBI was used to examine the lesions, and multiple images were captured for subsequent analysis. Images included both normal and abnormal areas for comparison. Image normalization was performed to ensure consistency in quality and size, with adjustments to brightness and contrast as necessary to optimize lesion visualization.

### Inclusion criteria:

Patients with confirmed or suspected early esophageal squamous cell neoplasia (ESCN) who underwent magnification endoscopic evaluation.

Availability of high-quality endoscopic images suitable for assessment of intrapapillary capillary loops (IPCLs).

Lesions were assessed using the JES microvessel classification system.

Patients aged 18 years or older.

### Exclusion criteria:

Poor-quality endoscopic images or incomplete image sets.

### JES IPCL classification

**FIGURE 1.**
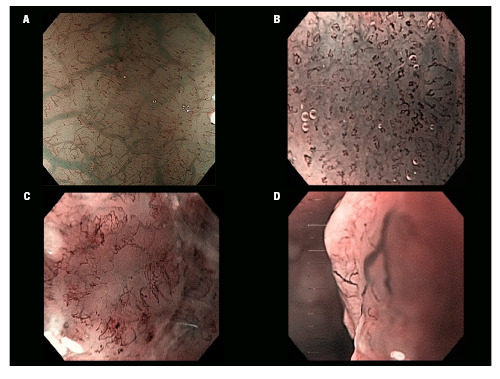
**A)** Magnification with NBI showing normal intrapapillary capillary loops (Type A); **B)** Abnormal microvessels with some irregularity and preserved loop-like formation (Type B1); **C)** Abnormal microvessels with severe irregularity and loss of loop-like formation (Type B2); **D)** Highly dilated abnormal vessels indicating deep submucosal invasion (Type B3).

The JES classification categorizes esophageal lesions based on IPCL morphology (Figure 1). Type A lesions reflect inflammatory changes, whereas types B1, B2, and B3 indicate dysplasia with increasing likelihood of deeper invasion, corresponding to EP/LPM, MM/SM1, and SM2 or deeper, respectively^8^. The auxiliary criteria evaluated included the presence of avascular areas (AVA) among others^9^.

### Evaluation process

The study involved a retrospective assessment of ME-NBI images of suspected ESCC. The best ME-NBI images showcasing the most characteristic pattern changes of 30 lesions were selected. Three online forms were created, each containing 10 cases, with each case comprising 2 to 5 images. A summary of the JES classification was provided at the beginning of each form. Ten Brazilian observers, divided into senior expert endoscopists and novice endoscopists, independently classified the 30 images without communication among them. They classified the lesions according to the JES IPCL classification and estimated the depth of mural invasion. If a lesion was not considered neoplastic, it was classified as inflammatory. The depth of mural invasion was estimated based on the IPCL pattern, auxiliary criteria, and the endoscopist’s subjective impression.

### Observers

Ten observers participated in this study: five seniors expert endoscopists and five novice residents. The examinations were performed by residents under the supervision of senior endoscopists.

- Senior expert endoscopists (EE): More than 10 years of experience in endoscopy and over 200 ME-NBI examinations of ESCC.
- Novice endoscopists (NE): Less than 3 years of experience in endoscopy and fewer than 50 ME-NBI examinations of ESCC. Sample size calculation to achieve a 95% intraclass correlation coefficient (ICC) with a sampling error of 0.20 for an expected kappa concordance coefficient of 0.50 was 30 cases. A standard deviation of 0.553 was assumed, and the statistical software PASS 14 (Power Analysis and Sample Size System) was used for the calculations. Statistical analysis interobserver agreement was assessed using Fleiss’ Kappa. Kappa values were interpreted according to Landis and Koch: values from 0 to 0.20 represent slight agreement, 0.21 to 0.40 fair agreement, 0.41 to 0.60 moderate agreement, 0.61 to 0.80 substantial agreement, and 0.81 to 1.00 almost perfect agreement^10^. The accuracy of the IPCL classification in predicting the depth of invasion was evaluated using sensitivity, specificity, positive predictive value (PPV), and negative predictive value (NPV). Histopathological results from resected specimens were used as the reference standard. Statistical analyses were performed using SPSS 20.0 and STATA 12 statistical software.

## RESULTS

### Global concordance vs JES classification

Agreement regarding IPCL classification was moderate across all observers, with a Kappa coefficient (K) of 0.455 (*P*<0.001). The experienced endoscopist (EE) group had a Kappa coefficient of 0.437 (*P*<0.001), while the novice endoscopist (NE) group had a Kappa coefficient of 0.483 (*P*<0.001). The highest concordances were observed for IPCL A (K=0.535, *P*<0.001) and IPCL B3 (K=0.547, *P*<0.001), whereas IPCL B2 showed only fair agreement (K=0.383, *P*<0.001). Table 1 provides a summary of the Kappa coefficient analysis.

**TABLE 1:** Global Kappa coefficient for all observers for JES classification (Interobserver).

	Total (N=10)			EE (N=5)			NE (N=5)		
	Kappa (k)	SE	*P*	Kappa (k)	SE	*P*	Kappa (k)	SE	*P*
**IPCL (JES classification)**	**0,455**	**0,017**	**<0.00**1	0,437	0,036	<0.001	0,483	0,035	<0.001
IPCL A	0,535	0,027	<0.001	0,527	0,058	<0.001	0,548	0,058	<0.001
IPCL B1	0,423	0,027	<0.001	0,409	0,058	<0.001	0,485	0,058	<0.001
IPCL B2	0,383	0,027	<0.001	0,397	0,058	<0.001	0,387	0,058	<0.001
IPCL B3	0,547	0,027	<0.001	0,478	0,058	<0.001	0,573	0,058	<0.001
**Auxiliary criteria**	**0,227**	**0,016**	<0.001	0,221	0,033	<0.001	0,199	0,034	<0.001
Reticular area	0,098	0,027	<0.001	0,126	0,058	0,014	0,054	0,058	0,173
AVA - small	0,118	0,027	<0.001	0,004	0,054	0,474	0,166	0,058	0,002
AVA - middle	0,140	0,027	<0.001	0,101	0,058	0,040	0,160	0,058	0,003
AVA - large	0,170	0,027	<0.001	0,101	0,058	0,040	0,140	0,058	0,008
Absent	0,403	0,027	<0.001	0,491	0,058	<0.001	0,292	0,058	<0.001
**Estimated invasion depth**	**0,439**	**0,017**	**<0.001**	0,374	0,035	<0.001	0,487	0,035	<0.001
Inflamatory	0,573	0,027	<0.001	0,556	0,058	<0.001	0,590	0,058	<0.001
Intramucosal	0,405	0,027	<0.001	0,409	0,058	<0.001	0,426	0,058	<0.001
Possibly superficial SM Invasion	0,365	0,027	<0.001	0,270	0,058	<0.001	0,413	0,058	<0.001
Deep SM invasion	0,491	0,027	<0.001	0,362	0,058	<0.001	0,590	0,058	<0.001

N=30 cases; SE: standard error; EE: experienced endoscopist; NE: novice endoscopist.

Regarding auxiliary criteria, the overall agreement was fair among all observers (K=0.227, *P*=0.016) and within the EE group (K=0.221, *P*=0.033), but slight in the NE group (K=0.199, *P*=0.034). The highest agree­ment was found for the absence of auxiliary criteria (K=0.403, *P*=0.027).

The agreement for the estimated depth of invasion was moderate across all observers (K=0.439, *P*<0.001), fair in the EE group (K=0.374, *P*<0.001), and moderate in the NE group (K=0.477, *P*<0.001). The highest agreement among all observers was for inflammatory lesions (K=0.573, *P*<0.001).

### Comparison with resected specimens

Among the 30 cases, 12 underwent endoscopic resection and five underwent surgical resection. The histopathological depth of invasion was classified as follows: three cases of high-grade dysplasia, three cases with EPm1 invasion, two with LPMm2, one with MMm3, and one with SM1sm1. In five cases, the invasion reached SM2sm3 or deeper, while two cases were classified as inflammatory lesions.

The overall accuracy of the JES classification for estimating the depth of invasion of SESCC was 47.5%, as shown in Table 2. The highest accuracy was from observer EE2 with 70.6% (95%CI 44.0-89.7). Observer NE4 had the lowest accuracy with 23.5% (95%CI 6.8-49.9).

**TABLE 2:** Distribution of global accuracy for JES classification.

Evaluators	Accuracy	
	%	95%CI
All	47.5	38.2-55.3
**Observers**		
EE1	52.9	27.8-77.0
EE2	70.6	44.0-89.7
EE3	58.8	32.9-81.6
EE4	47.1	23.0-72.2
EE5	41.2	18.4-67.1
NE1	52.9	27.8-77.0
NE2	47.1	23.0-72.2
NE3	35.3	14.2-61.7
NE4	23.5	6.8-49.9
NE5	41.2	18.4-67.1

CI95%: calculated via bootstrap based on 100 replicates; CI95%: confidence interval; EE: expericenced endoscopist; NE: novice endoscopist.

The diagnostic values of A, B1, B2, B3 IPCLs in the diagnosis of histologic depth of mural invasion are shown in Table 3. The assessment of IPCL classifications revealed varying degrees of diagnostic performance.

**TABLE 3:** Overall performance of the IPCL classification for the prediction of mural invasion.

	Sensitivity (95%CI)	Specificity (95% CI)	PPV (95%CI)	NPV (95% CI)	Accuracy (95%CI)
**A**	44.4 (30.0-50.0)	93.6 (89.3-97.3)	62.6 (38.3-85.8)	92.6 (90.7-93.6)	87.8 (83.5-91.8)
**B1**	41.6 (29.4-53.1)	76.2 (71.7-80.5)	57.9 (45.8-66.8)	60.4 (54.5-65.6)	59.9 (52.7-65.9)
**B2**	77.1 (62.6-95.0)	67.6 (59.7-76.0)	26.8 (19.0-36.6)	95.2 (92.1-98.9)	68.7 (60.6-77.3)
**B3**	46.4 (42.0-52.0)	92.0 (88.3-95.0)	71.8 (62.1-82.1)	80.5 (78.6-82.6)	78.6 (75.3-82.4)

CI95%: confidence interval calculated via boostrap based on 100 replicates; PPV: positive predictive value; VNP: negative predictive value.

## DISCUSSION

This study evaluated the interobserver agreement of the JES intrapapillary capillary loop (IPCL) classification for superficial squamous cell carcinoma (SESCC) of the esophagus in a Western cancer center. The overall interobserver agreement, indicated by kappa values, was moderately satisfactory (k=0.455, *P*<0.001). This suggests that while the JES IPCL classification is reproducible, there is room for improvement, especially in training and experience.

Our findings are consistent with previous studies indicating moderate to substantial interobserver agreement in the JES IPCL classification. Differently, a study by Mizumoto et al. analyzing 136 patients using magnifying endoscopy narrow band imaging (ME-NBI) reported better correspondence between interobserver and intraobserver agreements, highlighting the reproducibility and reliability of the classification among assessors with different experience levels^2^. Discrepancies with our results could be attributed to the use of static images, which may not fully capture the dynamic nature of IPCLs observed in real-time endoscopy.

The subgroup analysis of experienced (EE) and novice (NE) endoscopists revealed moderate agreement in both groups, different from previous studies that have shown higher concordance among experienced endoscopists^9^. This finding could be explained by the fact that our fellows (novice endoscopists) have a continuous exposure to magnifying-NBI endoscopy of ESCC. Our results suggest that the IPCL-JES classification is not difficult to reproduce even for less experienced endoscopists. However, the slight or fair agreement in auxiliary criteria of the JES classification reflects the difficulty in accurately characterizing these changes, particularly among less experienced endoscopists. This finding is corroborated by previous research indicating that dynamic observation and operator experience significantly impact diagnostic accuracy^4^.

The JES IPCL classification’s performance in predicting the depth of mural invasion showed greater specificities and positive predictive values for IPCL types A and B3. This indicates that while cases at the extreme positions in the spectrum of staging are easier to characterize, subtle changes in IPCLs pose a challenge. Expert endoscopists performed better than novices in predicting invasion depth, likely due to their ability to integrate subjective impressions with objective criteria^3^. Overdiagnosis of invasion depth could lead to unnecessary esophagectomies, with significant morbidity and mortality, whereas underdiagnosis might result in inadequate treatment. Thus, balancing these risks is crucial for optimizing patient outcomes. Our study emphasizes the need for training and experience in using the JES classification to minimize such diagnostic errors.

These study results parallel those of a recent multicenter study that evaluated the diagnostic accuracy and reproducibility of the JES microvessel classification in a Western cohort of 113 ESCN lesions^11^. In both studies, overall accuracy for predicting invasion depth was modest, 53% among Japanese experts, 52% for Western experts, and 44% for trainees in the multicenter series, compared to 47.5% in our Brazilian cohort. Likewise, interobserver agreement in both studies remained moderate, with Krippendorff’s alpha values between 0.58 and 0.64 among trained endoscopists and K=0.455 in our study. Notably, both analyses identified limited sensitivity for type B1 and B3 vessels, though specificity and negative predictive value for B3 vessels remained high, reinforcing their utility in excluding deep submucosal invasion^11^.

Our study has some limitations. Firstly, it was conducted in a single-center tertiary institution, which may limit the broader applicability of our findings. Secondly, the use of static images might not accurately represent real-time endoscopic assessments, potentially affecting the overall accuracy of the JES classification. Additionally, the sample size was not designed to comprehensively evaluate diagnostic accuracy. Future research should focus on larger, multicenter studies to validate our findings and explore the role of dynamic observation in enhancing diagnostic accuracy. Furthermore, incorporating new technologies, such as artificial intelligence (AI), could standardize the analysis and reduce observer variability. AI-assisted endoscopy has shown promise in improving diagnostic efficacy and reducing miss rates in detecting superficial esophageal lesions^7^. The effectiveness and cost-benefit of AI systems in real-world clinical settings need further assessment to confirm their potential utility.

In conclusion, our study found moderate interobserver agreement in the JES-IPCL classification of superficial ESCC among both expert and novice endoscopists, suggesting the potential utility of the JES-IPCL classification in the Western world, though the overall low performance indicates a need for extensive training. The findings highlight that the learning curve for accurately evaluating ME-NBI images using the JES classification may be longer than anticipated. Larger-scale analyses are warranted to assess its real accuracy in estimating the depth of invasion in SESCC. Endoscopy with magnification and IPCL evaluation remains an invaluable tool in the diagnosis and management of ESCC, providing critical diagnostic and prognostic information. Ongoing advancements in this field are expected to enhance the accuracy and reliability of IPCL evaluation, ultimately improving patient outcomes.
